# Corrected Version: Neutralization of RANTES and Eotaxin Prevents the Loss of Dopaminergic Neurons in a Mouse Model of Parkinson’s Disease

**DOI:** 10.33140/JCEI.11.03.04

**Published:** 2026-08-03

**Authors:** Goutam Chandra, Suresh B Rangasamy, Kalipada Pahan

**Affiliations:** Department of Neurological Sciences, Rush University Medical Center, Chicago, USA

**Keywords:** Parkinson’s Disease, MPTP, RANTES, Eotaxin, Dopamine, Dopaminergic Neurons, Microglia, T cells, Nigrostriatum, Neuroinflammation

## Abstract

Parkinson’s disease (PD) is second only to Alzheimer’s disease as the most common human neurodegenerative disorder. Despite intense investigation, no interdictive therapy is available for PD. Recent studies indicate that both innate and adaptive immune processes are active in PD. Accordingly, we found rapid increase in RANTES (regulated on activation, normal T cell expressed and secreted) and eotaxin, chemokines that are involved in T cell trafficking, in vivo in the substantia nigra pars compacta (SNpc) and the serum of 1-methyl-4-phenyl-1,2,3,6-tetrahydropyridine (MPTP)-intoxicated mice. RANTES and eotaxin were also upregulated in the SNpc of postmortem PD brains as compared to age-matched controls. Therefore, we investigated whether neutralization of RANTES and eotaxin could protect against nigrostriatal degeneration in MPTP-intoxicated mice. Interestingly, after peripheral administration, functional blocking antibodies against RANTES and eotaxin reduced the infiltration of CD4+ and CD8+ T cells into the nigra, attenuated nigral expression of proinflammatory molecules, and suppressed nigral activation of glial cells. These findings paralleled dopaminergic neuronal protection, normalized striatal neurotransmitters, and improved motor functions in MPTP-intoxicated mice. Therefore, we conclude that attenuation of chemokine-dependent adaptive immune response may be of therapeutic benefit for PD patients.

## 1. Introduction

In the 2016 article published in J. Biol. Chem, the second row of Figure 8B had an overlap with the third row [1]. Therefore, the article was withdrawn [1]. We have corrected this honest mistake in the current manuscript. Parkinson’s disease (PD) is the most common neurodegenerative movement disorder characterized by progressive loss of DA neurons in the ventral midbrain. Clinically, PD is characterized by tremor, bradykinesia, rigidity, and postural instability [2,3]. Pathologically, it is indicated by gliosis and progressive degeneration of the dopaminergic neurons associated with the presence of intracytoplasmic inclusions (Lewy bodies) in the substantia nigra pars compacta (SNpc) [4–6]. Although the etiology is poorly understood, PD is regulated by the adaptive arm of the immune system and a number of recent studies have shown the involvement of inflammatory T cells in nigrostriatal degeneration [7–13]. In a normal adult brain, the crosstalk between the peripheral immune system and the brain is transient, and there is no evidence that it leads to the brain inflammation. However, in chronic neurodegeneration, when disease becomes more widespread involving distant regions of the brain and periphery, a growing body of evidence suggests that the brain-resident microglia are not only activated, but happen to be “primed” by the systemic inflammation, leading to the exaggerated synthesis of pro-inflammatory molecules [14–21]. Earlier we have shown that microglial activation plays a critical role in the development of PD-related in mice and monkeys [15,18,22,23].

In numerous studies, we and others have also shown that microglial cells can be activated by the chronic infiltration of peripheral inflammatory T cells [24–26]. Accordingly, Brochard et al have shown that both CD8+ and CD4+ T cells significantly invade the SNpc in postmortem specimens from patients with PD and in MPTP-intoxicated mice [8]. They have also demonstrated that removal of CD4+, but not of CD8+, T cells in mice greatly reduced MPTP-induced nigrostriatal dopamine cell death [8]. According to Gendelman and colleagues, while Th17 cells exacerbate nigrostriatal dopaminergic neurodegeneration, regulatory T cells attenuate such neurodegeneration [9]. Although mechanisms leading to the infiltration of T cells into the CNS are poorly understood, recently we have seen marked upregulation of RANTES and eotaxin, chemokines that are involved in the infiltration of T cells and other immune cells, in vivo in the SNpc and the serum of MPTP-intoxicated monkey, suggesting that these chemokines may participate in nigrostriatal degeneration [10]. Accordingly, here, we demonstrate rapid upregulation of RANTES and eotaxin in nigra and serum of MPTP-intoxicated mice. Furthermore, we also delineate that RANTES and eotaxin are upregulated in the SNpc of postmortem PD brains as compared to age-matched controls and that functional blocking antibodies against RANTES and eotaxin protects against nigrostriatal degeneration in MPTP-intoxicated mice. These results suggest that neutralization of RANTES and eotaxin may be beneficial for PD patients.

## 2. Materials and Methods

- Reagents: Mouse RANTES and eotaxin ELISA kits were purchased from R&D Systems (Minneapolis, MN). Anti-CD3, CD4 and CD8 antibodies were purchased from eBioscience. Rabbit anti-TH antibody was purchased from Millipore. Anti-Iba-1 antibody was purchased from Abcam. Cy2- and Cy5-conjugated antibodies were obtained from Jackson Immuno Research Laboratories (West Grove, PA).
- Animals and MPTP Intoxication: Six- to eight-week old C57BL/6 mice were purchased from Harlan, Indianapolis, IN. Animal maintenance and experiments were in accordance with National Institutes of Health guidelines and were approved by the Institutional Animal Care and Use committee of the Rush University Medical Center, Chicago, IL. For acute MPTP intoxication, mice received four intraperitoneal (i.p.) injections of MPTP-HCl (18 mg/kg of free base; Sigma Chemical Co., St. Louis, MO) in saline at 2-h intervals [18,22–24,27]. Control animals received only saline.’
- Human Brain Tissue: Autopsy brain tissues from four male PD patients and four control subjects were obtained from the Rush PD Center Brain Bank. PD patients and control subjects did not differ significantly for their mean age at death (PD, 74 ± 3 years; control, 79 ± 18 years). The mean postmortem interval for PD and controls were 4.1 ± 0.8 and 10.9 ± 1.1 h, respectively.
- Treatment of MPTP-Intoxicated Mice with Neutralizing Antibodies against RANTES and Eotaxin: Azide-free neutralizing antibodies against RANTES/CCL5 and eotaxin/CCL11 were obtained from R&D Systems (Minneapolis, MN). After 2 h of the last injection of MPTP, mice were treated once with the combination of anti-RANTES Ab (20 μg/mouse) and anti-eotaxin Ab (20 μg/mouse) via i.p. injection. Antibodies were reconstituted in sterile PBS such a way that total injection volume remained at 100 μl per mouse. A group of MPTP-intoxicated mice also received IgG (40 μg/mouse) as control via i.p. injection.
- Semi-Quantitative RT-PCR Analysis: Total RNA was isolated from nigra using Ultraspec-II RNA reagent (Biotecx Laboratories, Inc., Houston, TX) following the manufacturer’s protocol. To remove any contaminating genomic DNA, total RNA was digested with DNase. RT-PCR was carried out as described earlier [22,28,29] using a RT-PCR kit (Clontech, Mountain View, CA) and following primers:
  - iNOS: Sense: 5’-CCCTTCCGAAGTTTCTGGCAGCAGC-3’
  - Antisense: 5’-GGCTGTCAGAGCCTCGTGGCTTTGG3’
  - IL-1β: Sense: 5’-CTCCATGAGCTTTGTACAAGG-3’
  - Antisense: 5’-TGCTGATGTACCAGTTGGGG-3’
  - IL-6: Sense: 5’-GACAACTTTGGCATTGTGG-3’
  - Antisense: 5’-ATGCAGGGATGATGTTCTG-3’
  - TNFα:Sense: 5’-TTCTGTCTACTGAACTTCGGGGTGATCGGTCC-3’
  - Antisense: 5’-GTATGAGATAGCAAATCGGCTGACGGTGTGGG-3’
  - RANTES: Sense: 5’-ATACGCTTCCCTGTCATCGC-3’
  - Antisense: 5’-TTGGGTTTCGTGGTCGAGAG-3’
  - Eotaxin: Sense: 5’-AGCTAGTCGGGAGAGCCTAC-3’
  - Antisense: 5’-AAGGAAGTGACCGTGAGCAG-3’
  - CD11b: Sense: 5’- GTGAGGATTCCTACGGGACCCAGGT-3’
  - Antisense: 5’-GGCGTACTTCACAGGCAGCTCCAAC-3’
  - GFAP: Sense: 5’- GGCGCTCAATGCTGGCTTCA-3’
  - Antisense: 5’- TCTGCCTCCAGCCTCAGGTT-3’
  - GAPDH: Sense: 5’-GGTGAAGGTCGGTGTGAACG3’
  - Antisense: 5’-TTGGCTCCACCCTTCAAGTG-3’
- Real-Time PCR Analysis: DNase-digested RNA was analyzed by real-time PCR in the ABI-Prism7700 sequence detection system (Applied Biosystems, Foster City, CA) as described earlier using TaqMan Universal Master mix and optimized concentrations of FAM-labeled probes and primers [22,28–30]. Data were processed using the ABI Sequence Detection System 1.6 software. Western blot Analysis: Immunoblot analysis for RANTES, eotaxin, iNOS, IL-1β, GFAP, Iba-1, and TH was carried out as described earlier [31–33]. Briefly, cell homogenates were electrophoresed, proteins were transferred onto a nitrocellulose membrane, and bands were visualized with an Odyssey infrared scanner after immunolabeling with respective primary antibodies followed by infra-red fluorophore-tagged secondary antibody (Invitrogen).
- Immunohistochemistry and Quantitative Morphology: Seven days after MPTP intoxication, mice were sacrificed and their brains fixed, embedded, sectioned (30 μm thick), and processed for tyrosine hydroxylase (TH) and thionic staining as described previously [23,34]. Total numbers of TH- and Nissl-stained neurons in SNpc were counted stereologically with STEREO INVESTIGATOR software (Micro Brightfield, Williston, VT) by using an optical fractionator [23,34]. Quantitation of striatal TH immunostaining was performed as described [23,34]. Optical density measurements were obtained by digital image analysis (Scion, Frederick, MD). Striatal TH optical density reflected dopaminergic fiber innervation.
  For immunofluorescence staining on fresh frozen nigral sections isolated from mice after 1 d of MPTP insult, goat anti-mouse RANTES (1:100), rat anti-mouse eotaxin (1:100), rabbit anti-mouse Iba1 (1:100), goat anti-mouse GFAP (1:100), mouse anti-mouse CD4 (1:100), and mouse anti-mouse iNOS (1:250) were used. The samples were mounted and observed under an Olympus IX81 fluorescence microscope. Counting analysis was performed using Olympus microsite V software with the help of touch counting module. After acquiring images under 20 X objective lens, images were further analyzed as follows. Before counting cells, entire Image area was calibrated with the help of a rectangular box available in the touch counting panel. Once the area of the image was measured, touch counting program was applied to count number of fluorescent signals using simple mouse click method. Next, the total number of signals in a given area was divided by the total area of the image and presented as number of cells per square millimeter unit.
- HPLC Analysis for Measurement of Striatal Dopamine and its Metabolite Levels: Striatal level of dopamine, DOPAC (3, 4-dihydroxyphenylacetic acid) and HVA (homovanillic acid) was quantified as described earlier [15,18,23,24,34]. Briefly, mice were sacrificed by cervical dislocation after 7 days of MPTP intoxication and their striate were collected and immediately frozen in dry ice and stored at −80C until analysis. On the day of the analysis, tissues were sonicated in 0.2M perchloric acid containing isoproterenol and resulting homogenates were centrifuged at 20,000 x g for 15 min at 4C. After pH adjustment and filtration, 10μl of supernatant was injected onto an Eicompak SC-3ODS column (Complete Stand-Alone HPLC-ECD System EiCOMHTEC-500 from JM Science Inc., Grand Island, NY) and analyzed following manufacturer’s protocol.
- Behavioral Analyses: Three types of behavioral experiments were conducted. These included an open field experiment for locomotor activity, a pole test for bradykinesia and a rotorod experiment for feet movement as described earlier [18,22–24,35]. Locomotor activity was measured after 7 d of the last dose of MPTP injection in a Digi scan Monitor (Omnitech Electronics, Inc., Columbus, OH). This Digi scan Monitor records stereotypy and rearing, behaviors that are directly controlled by striatum, as well as other basic locomotion parameters, such as horizontal activity, total distance traveled, number of movements, movement time, rest time, mean distance, mean time, and center time. Before any insult or treatment, mice were placed inside the Digi scan Infra-red Activity Monitor for 10 min daily and on rotarod for 10 min daily for 3 consecutive days to train them and record their baseline values. Briefly, animals were removed directly from their cages and gently placed nose first into a specified corner of the open-field apparatus and after release, data acquisition began at every 5 min interval. DIGISCAN software was used to analyze and store horizontal and vertical activity data, which were monitored automatically by infra-red beams. Bradykinesia was measured by the time to turn head-down and completely descend a wooden pole wrapped in cloth tape. Briefly, mice were acclimatized to the pole (1 cm diameter, 40 cm height) over 3 trials of 120 s each. Each trial was separated by 60 s and during behavioral testing, each mouse was tested thrice. In rotarod, the feet movement of the mice was observed at different speeds. To eliminate stress and fatigues, mice were given a 5-min rest interval.
- Statistical Analysis: All values are expressed as means ± SEM. Differences among means were analyzed by one-way ANOVA or Kruskal-Wallis test (comparison among all four groups) and post-hoc pair-wise comparison. In other cases, two sample t tests were also used to compare control vs MPTP and MPTP vs antibody.

## 3. Results

### 3.1. Rapid Induction of RANTES and Eotaxin in Nigra and Serum of MPTP-Intoxicated Mice

**Figure 1:**
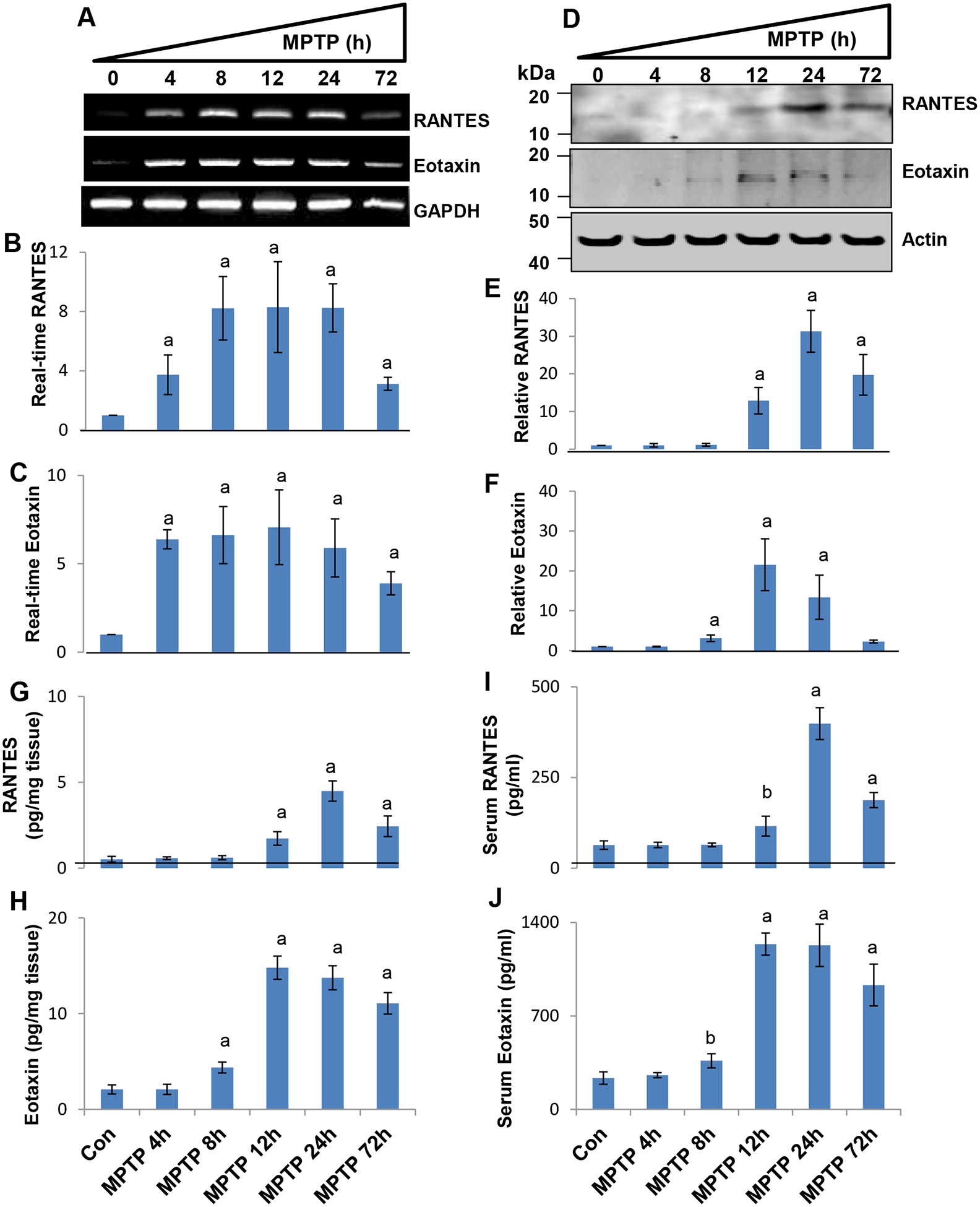
Rapid upregulation of RANTES and eotaxin in nigra and serum of MPTP-intoxicated mice. Male C57/BL6 mice (6–8-week-old) were insulted with 20 mg/kg body wt MPTP (four injections at every 2 h interval). After 4, 8, 12, 24, and 72 h of MPTP intoxication, the mRNA expression of RANTES and eotaxin in nigra was monitored by semi-quantitative RT-PCR (A) and real-time PCR (B, RANTES; C, eotaxin). The protein expression of RANTES and eotaxin was monitored by Western blot (D). Actin was run as control. Bands were scanned and values (E, RANTES/Actin; F, Eotaxin/Actin) are presented as relative to control. Levels of RANTES and eotaxin were also measured in nigral homogenates by ELISA (G, RANTES; H, eotaxin). Levels of RANTES and eotaxin were also measured in serum by ELISA (I, RANTES; J, eotaxin). Results are mean + SEM of four mice (n=4) per group. ap < 0.001 vs control; bp < 0.05 vs control.

To investigate the role of RANTES and eotaxin in the loss of invaluable dopaminergic neurons in MPTP-intoxicated mice, first, we examined whether the expression of these chemokines was induced in midbrains of affected mice. It is evident from Figure 1A that MPTP intoxication led to time-dependent induction of RANTES and eotaxin mRNA expression in the SNpc. This induction was evident as early as 4 h of MPTP insult (Figure 1A). However, the expression of RANTES and eotaxin decreased at 72 h of MPTP intoxication (Figure 1A). These results were confirmed by real-time PCR (Figure 1B–C). Similarly, Western blot results showed the induction of RANTES and eotaxin proteins in the nigra (Figure 1D–F). Although RANTES was visible from 12 h of MPTP insult, significant increase in eotaxin was observed at 8 h (Figure 1E–F). These results were also corroborated by ELISA of RANTES and eotaxin in nigral homogenates (Figure 1G–H). Next, we monitored the levels of these chemokines in serum. While increase in RANTES was visible in serum from 12 h of MPTP intoxication and maximum at 24 h, eotaxin increase was prominent from 8 h and maximum at 12 h (Figure 1I–J).

### 3.2. Microglia in the Nigra of MPTP-Intoxicated Mice and Postmortem PD Brains Express RANTES and Eotaxin

**Figure 2:**
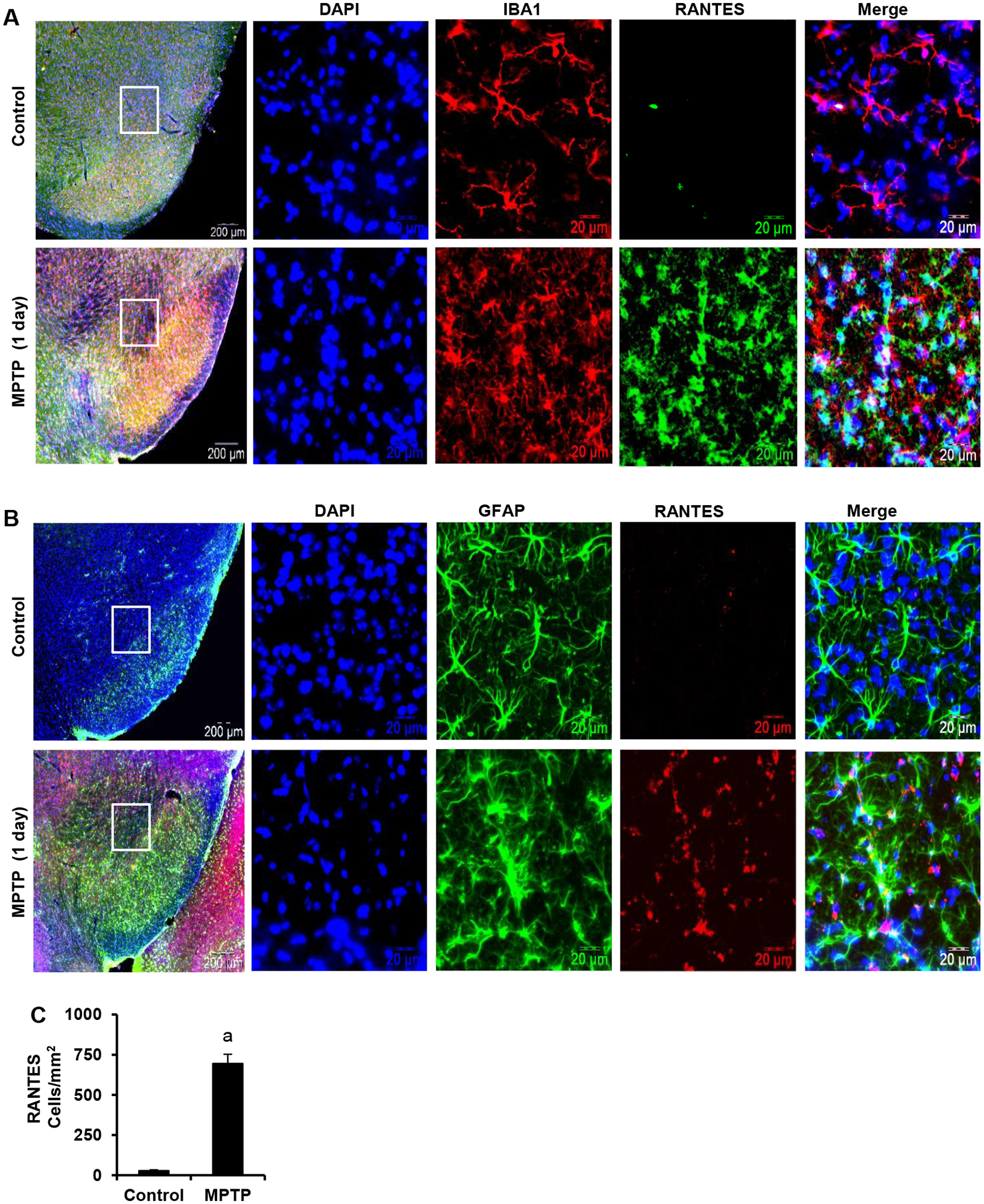
Glial expression of RANTES in the SNpc of MPTP-intoxicated mice. A) Male C57/BL6 mice (6–8-week-old) were insulted with 20 mg/kg body wt MPTP (four injections at every 2 h interval). After 1 d, nigral sections were double-labeled (A, Iba-1 & RANTES; B, GFAP & RANTES). Cells positive for RANTES (C) were counted in two nigral sections (two images per slide) of each of five mice (n=5) per group as described under “Materials and Methods”. ap<0.001 vs. control.

**Figure 3:**
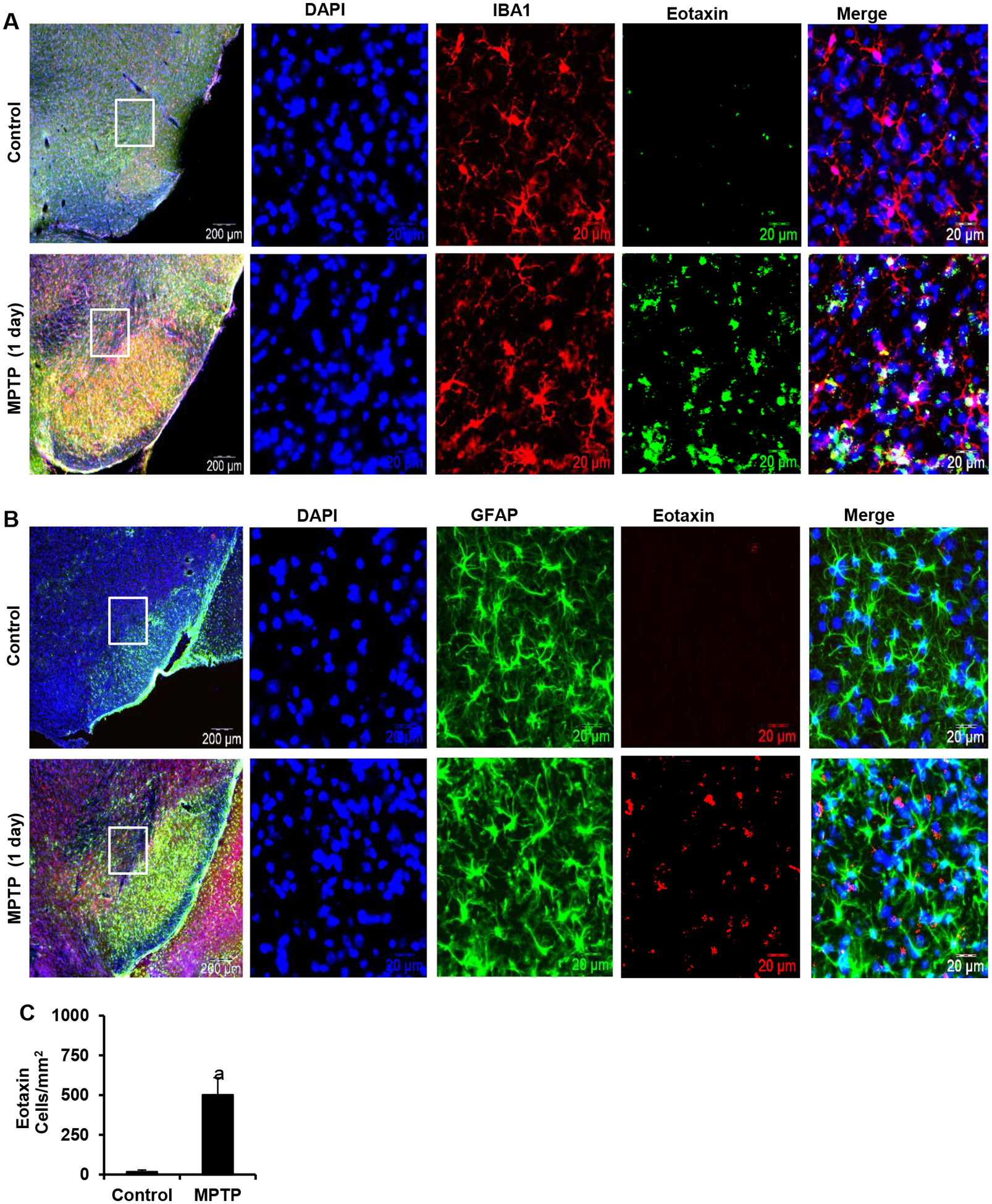
Induction of eotaxin in the SNpc of MPTP-intoxicated mice. A) Male C57/BL6 mice (6–8 week old) were insulted with 20 mg/kg body wt MPTP (four injections at every 2 h interval). After 1 d, nigral sections were double-labeled (A, Iba-1 & eotaxin; B, GFAP & eotaxin). Cells positive for eotaxin (C) were counted in two nigral sections (two images per slide) of each of five mice (n=5) per group. ap<0.001 vs. control.

Since MPTP intoxication induced the level of RANTES and eotaxin in the nigra, next, we were interested to identify the cell type that produced these chemokines in the nigra. Recently chronic microglial activation is becoming a hallmark of different neurodegenerative disorders including PD [22,23,36–38]. Therefore, we examined if microglia were capable of producing these chemokines in the nigra of MPTP-intoxicated mice. After 24 h of the last injection of MPTP, nigral sections were double-labeled for Iba-1 and RANTES. As evident from Figure 2A–C, MPTP intoxication led to marked induction of RANTES in the nigra and most of these RANTES signals colocalized with Iba-1 (Figure 2A). In addition, some RANTES signals also colocalized with GFAP-positive astroglia (Figure 2B). Similarly, immunofluorescence analysis also reveals marked increase in eotaxin in the nigra of MPTP-intoxicated mice (Figure 3A–C). Similar to RANTES, eotaxin also mostly colocalized with Iba-1-positive microglia (Figure 3A) and partly with GFAP-positive astroglia (Figure 3B).

**Figure 4:**
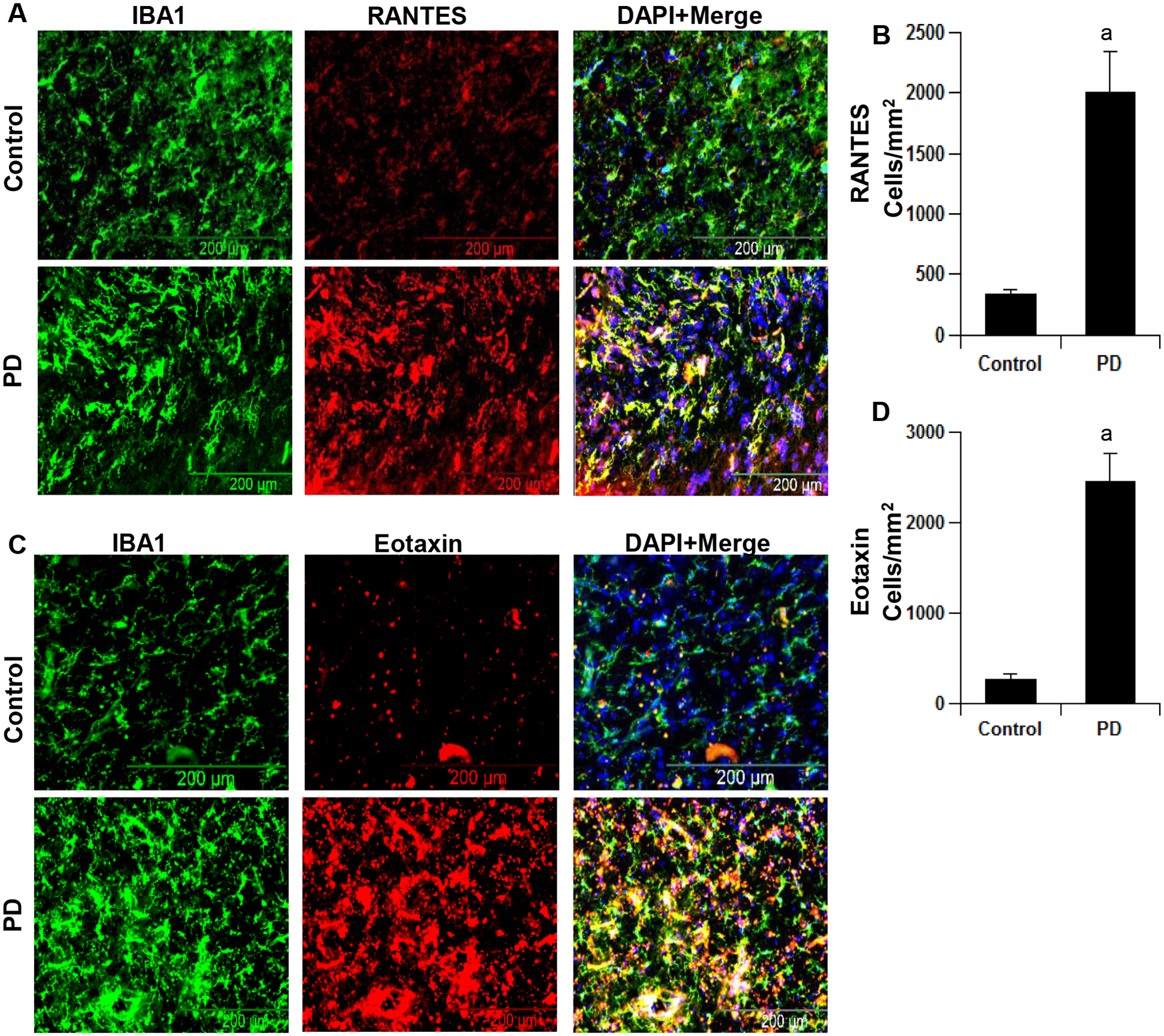
Presence of RANTES and eotaxin in the SNpc of postmortem PD brains. Midbrain sections of postmortem PD brains and age-matched controls were double-labeled (A, Iba-1 & RANTES; B, Iba-1 & eotaxin). Cells positive for RANTES (C) and eotaxin (D) were counted in two nigral sections (two images per slide) of each of four brains (n=4) per group. ap<0.001 vs. control.

Next, to understand the role of RANTES and eotaxin in nigrostriatal degeneration in PD, nigral sections from postmortem PD brains and age-matched individuals were immunolabeled for RANTES and eotaxin. Since microglia is the major cell type in the nigra of MPTP-intoxicated mice that express these chemokines, sections were double-labeled for RANTES/eotaxin and Iba-1. Levels of both RANTES and eotaxin were markedly higher in the nigra of PD brain compared to age-matched controls (Figure 4A–D). We also noticed greater Iba-1 expression (microglial activation) in the nigra of PD compared to age-matched controls (Figure 4A & 4C). Iba-1-positive cells were also positive for both RANTES (Figure 4A) and eotaxin (Figure 4C) in the nigra of PD subjects.

### 3.3. Functional Blocking Antibodies against RANTES and Eotaxin Suppresses the Infiltration of T Cells into the Nigra and Attenuates the Expression of Proinflammatory Molecules in the Nigra of MPTP-Intoxicated Mice

**Figure 5:**
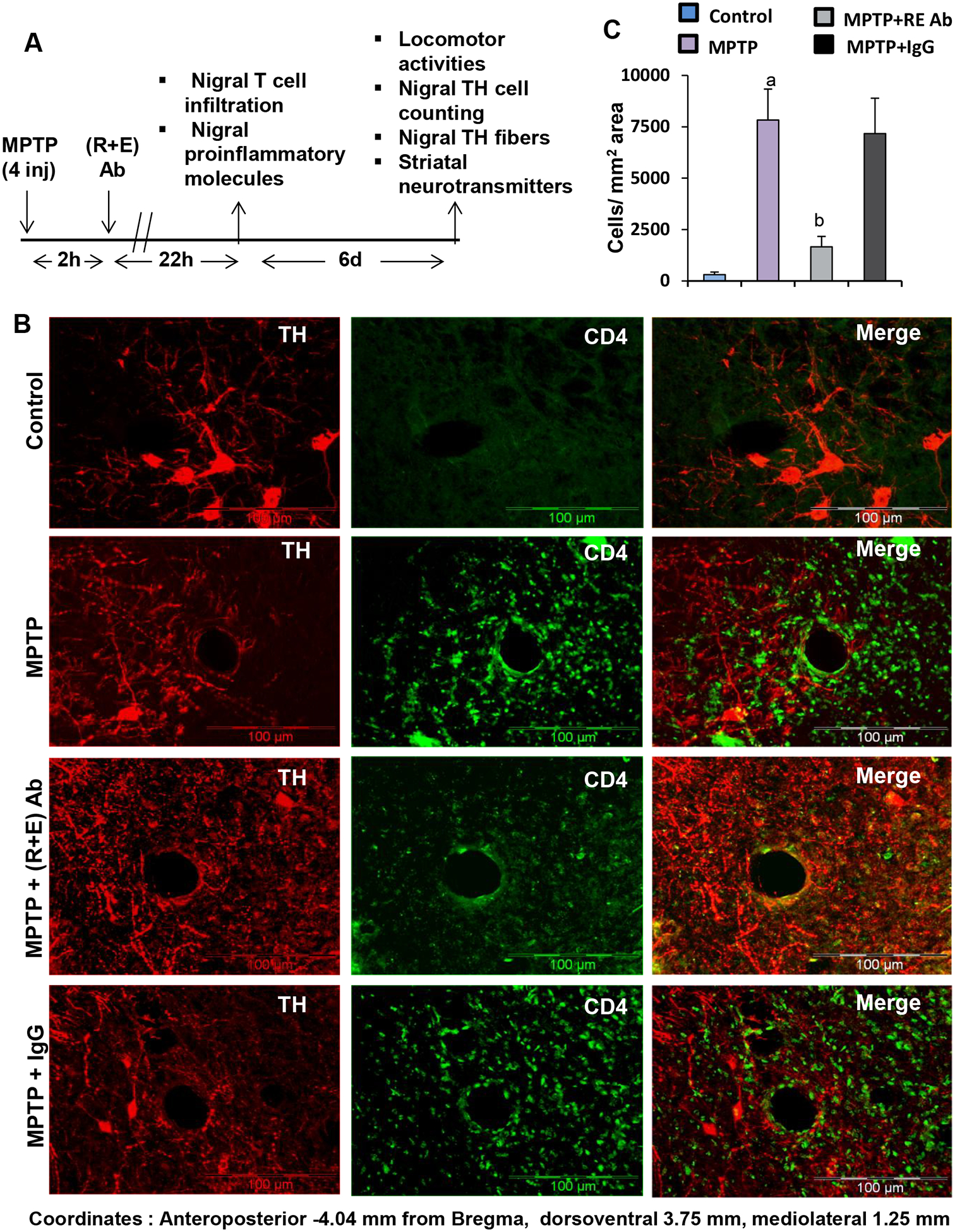
Functional blocking antibodies against RANTES and eotaxin inhibit the infiltration of CD4+ T cells into the nigra of MPTP-intoxicated mice. A) Schematic presentation of treatment of MPTP-intoxicated mice with antibodies and related experiments. B) Male C57/BL6 mice (6–8-week-old) were insulted with 20 mg/kg body wt MPTP (four injections at every 2 h interval). After 2 h of the last injection of MPTP, animals were treated with the combination of 20 μg/mouse anti-RANTES Ab and 20 μg/mouse anti-eotaxin Ab via i.p. injection. After 1 d of the last injection of MPTP, ventral midbrain sections (Coordinates: Anteroposterior −4.04 mm from Bregma, dorsoventral 3.75 mm, mediolateral 1.25 mm) were double-labeled for CD4 and TH. C) CD4-positive cells were counted in two nigral sections (two images per slide) of each of five mice (n=5) per group. ap<0.001 vs. control; bp<0.001 vs. MPTP.

**Figure 6:**
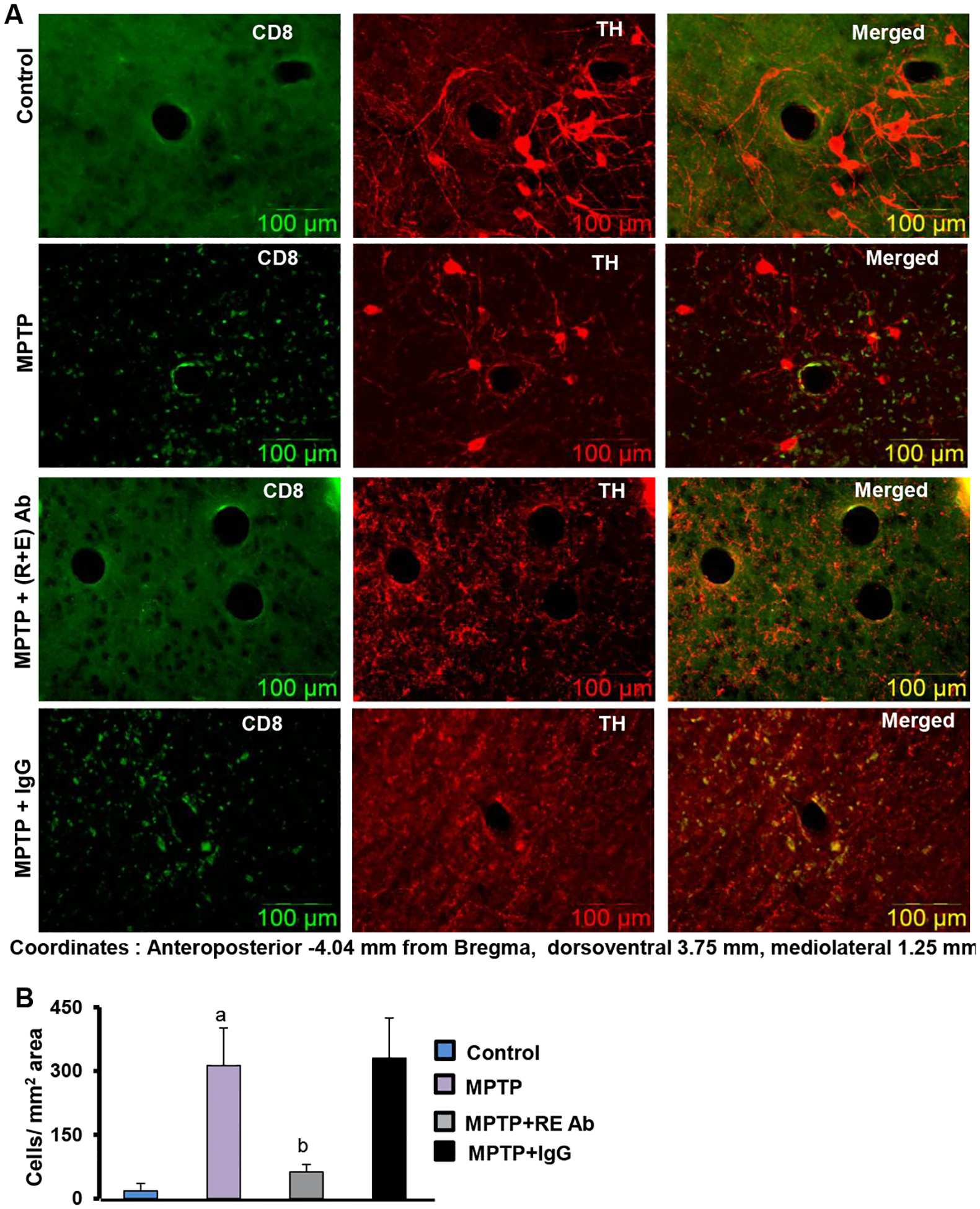
Functional blocking antibodies against RANTES and eotaxin inhibit the infiltration of CD8+ T cells into the nigra of MPTP-intoxicated mice. A) Male C57/BL6 mice (6–8-week-old) were insulted with 20 mg/kg body wt MPTP (four injections at every 2 h interval). After 2 h of the last injection of MPTP, animals were treated with the combination of 20 μg/mouse anti-RANTES Ab and 20 μg/mouse anti-eotaxin Ab via i.p. injection. After 1 d of the last injection of MPTP, ventral midbrain sections (Coordinates: Anteroposterior −4.04 mm from Bregma, dorsoventral 3.75 mm, mediolateral 1.25 mm) were double-labeled for CD8 and TH. C) CD8-positive cells were counted in two nigral sections (two images per slide) of each of five mice (n=5) per group. ap<0.001 vs. control; bp<0.001 vs. MPTP.

Since we observed rapid increase in RANTES and eotaxin in the serum of MPTP-intoxicated mice, to understand the role of these chemokines in nigrostriatal degeneration, mice were treated once with the combination of functional blocking antibodies against both RANTES and eotaxin via i.p. injection (Figure 5A). Chemokines like RANTES and eotaxin are known to induce the migration and homing of inflammatory lymphoid cells such as T cells and monocytes in the site of inflammation. Because substantia nigra is a primary target of neurodegeneration in PD, we determined whether MPTP intoxication induced the infiltration of inflammatory T cells in the nigra. Our dual immunofluorescence analyses of CD4 (green) and tyrosine hydroxylase (TH) (red) clearly displayed a typical CD4-immunoreactive inflammatory cuffing in the nigra of MPTP-intoxicated, but not control, mice (Figure 5B–C). This is in consistent to that observed in the nigra of PD patients [8]. Recently we have demonstrated infiltration of CD8+ T cells into the nigra of hemi parkinsonian monkeys [10]. Therefore, we also analyzed infiltration of CD8+ T cells and found CD8+ inflammatory cuffing in the nigra of MPTP-insulted mice (Figure 6A–B). However, as compared to CD4+ T cells, nigral infiltration of CD8+ cells were much less in MPTP-intoxicated mice (Figure 5B–C & Figure 6A–B). Nevertheless, treatment with neutralizing antibodies against RANTES and eotaxin strongly suppressed the infiltration of both CD4+ (Figure 5B–C) and CD8+ (Figure 6A–B) T cells in the nigra of MPTP-intoxicated mice. These results were specific as normal IgG had no such inhibitory effect (Figures 5–6). These results suggest that the infiltration of peripheral lymphocytes into the nigra of MPTP-insulted mice depends on RANTES and eotaxin.

**Figure 7:**
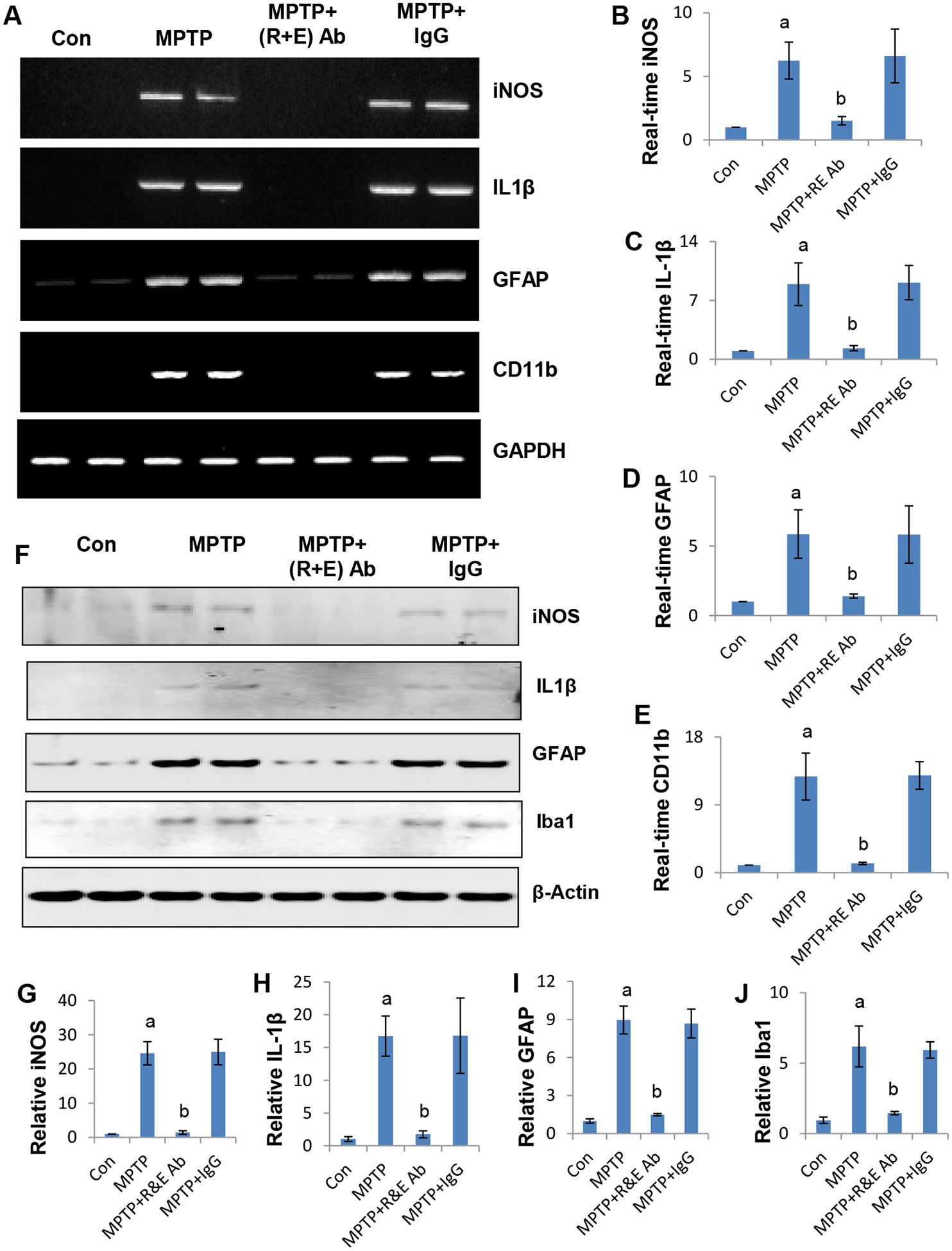
Neutralization of RANTES and eotaxin reduces the expression of proinflammatory molecules in the nigra of MPTP-intoxicated mice. Male C57/BL6 mice (6–8-week-old) were insulted with 20 mg/kg body wt MPTP (four injections at every 2 h interval). After 2 h of the last injection of MPTP, animals were treated with the combination of 20 μg/mouse anti-RANTES Ab and 20 μg/mouse anti-eotaxin Ab via i.p. injection. After 1 d of the last injection of MPTP, the mRNA expression of iNOS, IL-1β, GFAP, and CD11b was monitored in the nigra by RT-PCR (A) and real-time PCR (B, iNOS; C, IL-1β; D, GFAP; E, CD11b). Results are mean + SEM of four mice (n=4) per group. ap < 0.001 vs control; bp < 0.001 vs MPTP. After 1 d of the last injection of MPTP, the protein expression of iNOS, IL-1β, GFAP, and Iba1 was monitored in the nigra by Western blot (F). Actin was run as control. Bands were scanned and values (G, iNOS/Actin; H, IL-1β/Actin; I, GFAP/Actin; J, Iba1/Actin) are presented as relative to control. Results are mean + SEM of four mice (n=4) per group. ap < 0.001 vs control; bp < 0.001 vs MPTP.

**Figure 8:**
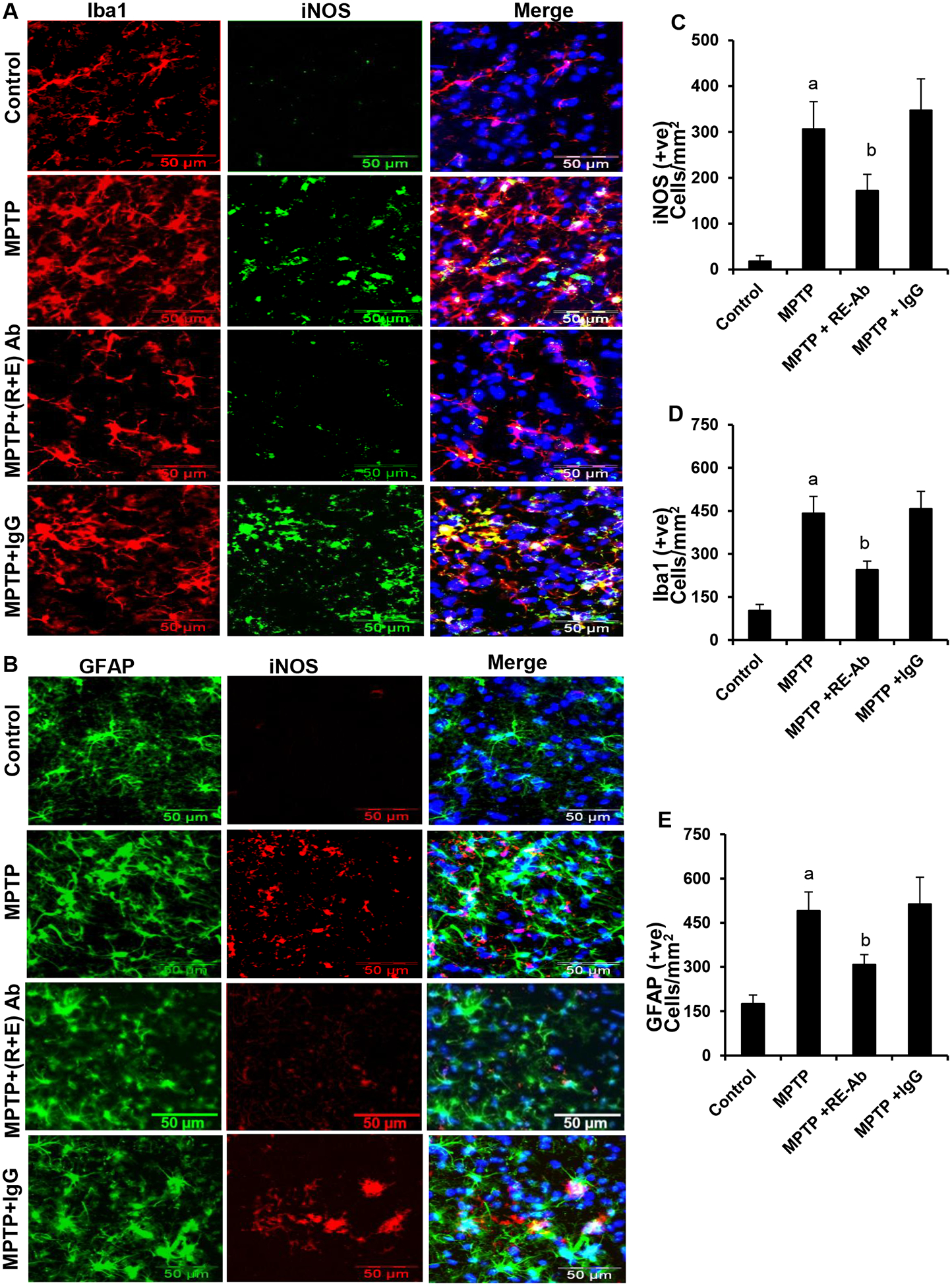
Neutralization of RANTES and eotaxin decreases glial activation in the nigra of MPTP-intoxicated mice. Male C57/BL6 mice (6–8-week-old) were insulted with 20 mg/kg body wt MPTP (four injections at every 2 h interval). After 2 h of the last injection of MPTP, animals were treated with the combination of 20 μg/mouse anti-RANTES Ab and 20 μg/mouse anti-eotaxin Ab via i.p. injection. After 1 d of the last injection of MPTP, nigral sections were double-labeled (A, Iba-1 & iNOS; B, GFAP & iNOS). Cells positive for iNOS (C), Iba-1 (D) and GFAP (E) were counted in two nigral sections (two images per slide) of each of five mice (n=5) per group. ap<0.001 vs. control; bp<0.001 vs. control.

Infiltration of inflammatory T cells into the site of injury eventually triggers the production of a wide range of proinflammatory molecules [8,11,24,36]. Because neutralizing antibodies against RANTES and eotaxin inhibited the infiltration of T cells in vivo in the nigra of MPTP-intoxicated mice, we examined whether these antibodies were able to suppress the expression of various proinflammatory molecules in the nigra. As shown by semi-quantitative RT-PCR (Figure 7A) and quantitative real-time PCR (Figure7B–E) experiments, MPTP intoxication led to marked increase in mRNA expression of iNOS, IL-1β, GFAP (astroglial marker), and CD11b (microglial marker) in the midbrain. However, neutralizing antibodies against RANTES and eotaxin, but not control IgG, strongly inhibited MPTP-induced expression of iNOS (Figure 7A&B), IL-1β (Figure 7A&C), GFAP (Figure 7A&D), and CD11b (Figure 7A&E) mRNAs in vivo in the nigra. Similarly, Western blot results also show increase in iNOS, IL-1β, GFAP, and Iba1 in the nigra by MPTP insult and attenuation of these proinflammatory markers (Figure 7F–J) by treatment with neutralizing antibodies against RANTES and eotaxin. Double-label immunofluorescence analysis also shows that MPTP intoxication led to marked increase in nigral iNOS protein expression and that this iNOS colocalized strongly with Iba1-positive microglia (Figure 8A) and partly with GFAP-positive astroglia (Figure 8B). Similar to mRNA and Western blot results, treatment of MPTP-intoxicated mice with neutralizing antibodies against RANTES and eotaxin, but not control IgG, led to the suppression of iNOS protein (Figure 8A–C). Recently glial activation is being considered as a pathological hallmark in PD and other neurodegenerative disorders [22,23,36,37]. As evident from immunofluorescence analysis of Iba1 and GFAP in nigral sections, MPTP intoxication led to increase in nigral Iba1 and GFAP protein expression and neutralizing antibodies against RANTES and eotaxin suppressed MPTP-induced expression of Iba1 (Figure 8A&D) and GFAP (Figure 8A&E). These results suggest that neutralization of RANTES and eotaxin suppresses the expression of proinflammatory molecules and reduces glial activation in the nigra of MPTP-intoxicated mice.

### 3.4. Functional Blocking Antibodies against RANTES and Eotaxin Protect against MPTP-Induced Neurodegeneration

**Figure 9:**
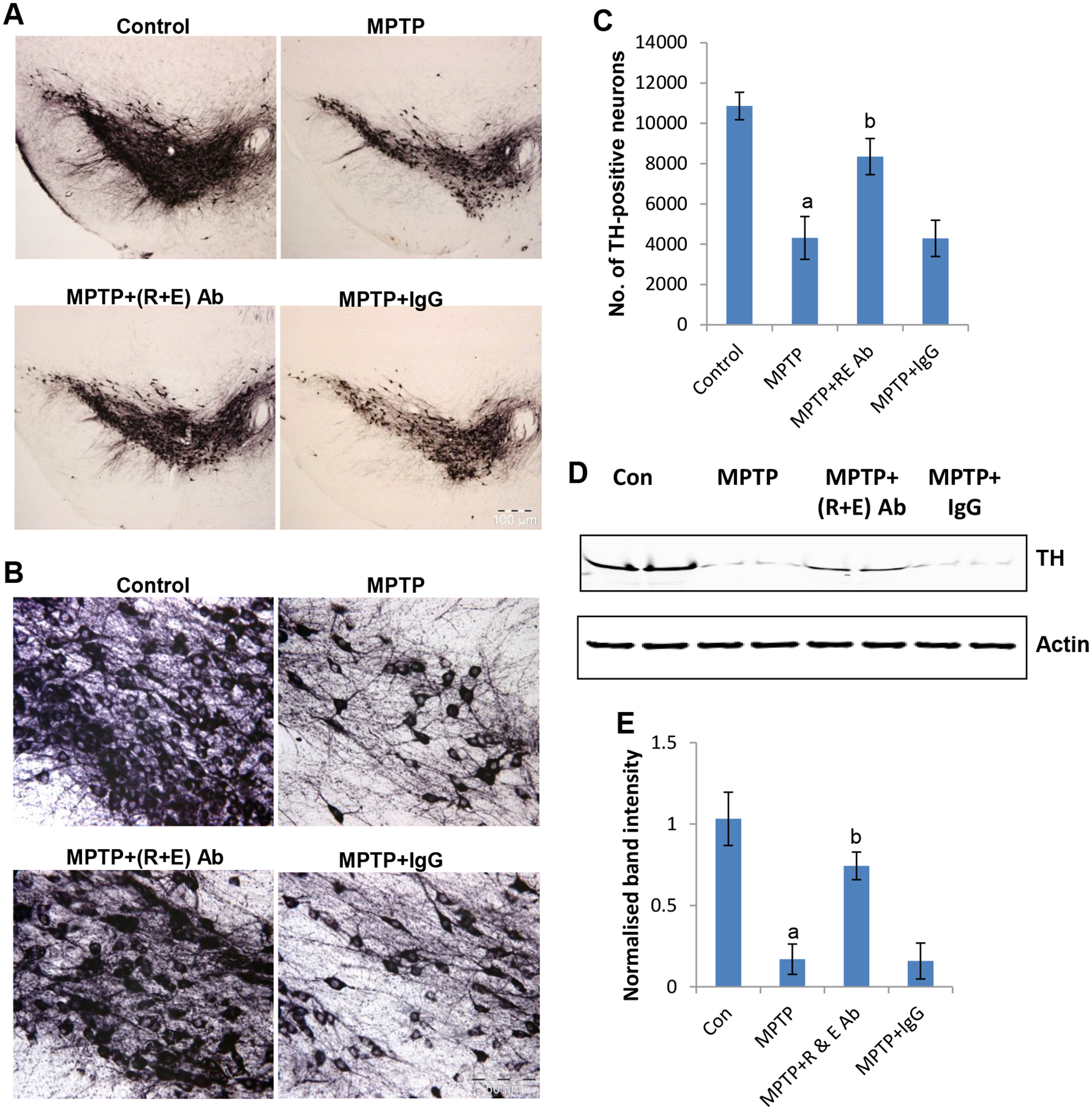
Neutralization of RANTES and eotaxin protects dopaminergic neurons in the nigra of MPTP-intoxicated mice. Male C57/BL6 mice (6–8-week-old) were insulted with 20 mg/kg body wt MPTP (four injections at every 2 h interval). After 2 h of the last injection of MPTP, animals were treated with the combination of 20 μg/mouse anti-RANTES Ab and 20 μg/mouse anti-eotaxin Ab via i.p. injection. After 7 d of the last injection of MPTP, nigral sections were stained for TH (A). Magnified image TH-stained SNpc (B). TH neurons were counted by stereology using the STEREO INVESTIGATOR software (C). Results are mean + SEM of five mice (n=5) per group. ap < 0.001 vs control; bp < 0.001 vs MPTP. Nigral homogenates were immunoblotted for TH (D). Actin was run as control. Bands were scanned and values (TH/Actin) are presented as relative to control (E). Results are mean + SEM of four mice (n=4) per group. ap < 0.001 vs control; bp < 0.001 vs MPTP.

Since neutralization of RANTES and eotaxin inhibited glial activation and associated neuroinflammation in the nigra of MPTP-intoxicated mice, next, we investigated if functional blocking antibodies against RANTES and eotaxin protected the nigrostriatum from MPTP insult. Mice were treated once with functional blocking antibodies against RANTES and eotaxin 2 h after the last injection of MPTP and seven days after the last injection of MPTP, status of nigral TH neurons and striatal TH fibers were monitored. MPTP-intoxication led to approximately 65% loss of SNpc TH-positive neurons (Figure 9A–B) compared with saline-injected controls. However, in MPTP-injected mice treated with functional blocking antibodies against RANTES and eotaxin, the reduction in SNpc TH-positive neurons was about 22% (Figure 9A–B). On the other hand, no such protective effects were seen in MPTP-intoxicated mice that were treated with control IgG (Figure 9A–B). Results were also corroborated by TH Western blot data of nigral homogenates (Figure 9C–D).

**Figure 10:**
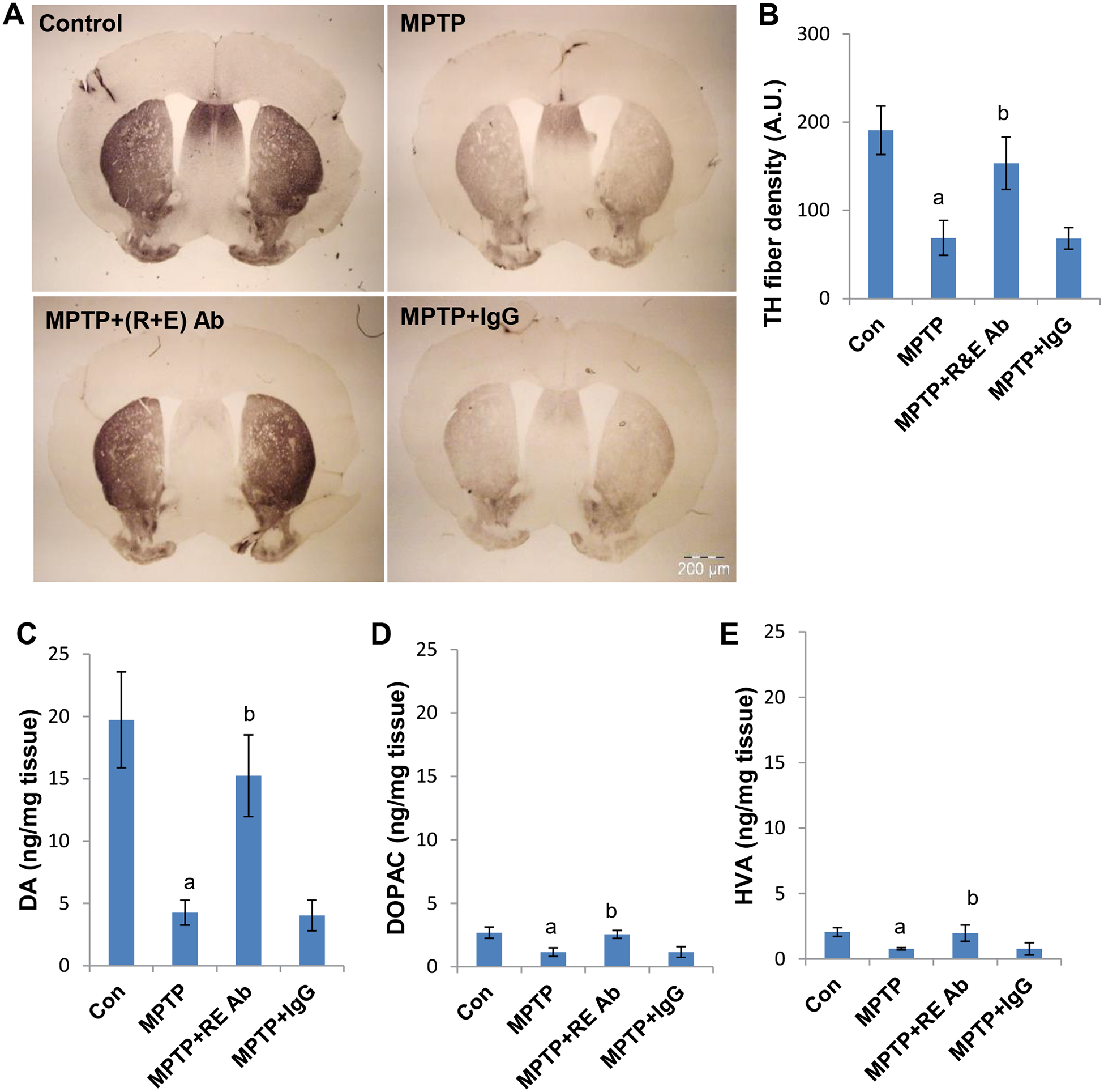
Neutralization of RANTES and eotaxin protects TH fibers and restores neurotransmitters in the striatum of MPTP-intoxicated mice. Male C57/BL6 mice (6–8-week-old) were insulted with 20 mg/kg body wt MPTP (four injections at every 2 h interval). After 2 h of the last injection of MPTP, animals were treated with the combination of 20 μg/mouse anti-RANTES Ab and 20 μg/mouse anti-eotaxin Ab via i.p. injection. After 7 d of the last injection of MPTP, striatal sections were stained for TH (A) followed by quantification of TH-positive fibers (B). Concentrations of dopamine (C), DOPAC (D) and HVA (E) were measured in the striatum by HPLC. Results are mean + SEM of five mice (n=5) per group. ap < 0.001 vs control; bp < 0.001 vs MPTP.

Similar to the loss of nigral TH neurons, MPTP-intoxication led to approximately 68% reduction of striatal TH ODs (Figure 10A–B) compared with saline-injected controls. Again, marked protection of striatal TH fibers was noted in MPTP-injected mice treated with neutralizing antibodies against RANTES and eotaxin (Figure 10A–B). To determine whether neutralization of RANTES and eotaxin protects against biochemical deficits caused by MPTP, we quantified the level of DA, DOPAC and HVA in the striata 7 days after the MPTP treatment. MPTP intoxication led to marked decrease in striatal DA (Figure 10C), DOPAC (Figure 10D) and HVA (Figure 10E) compared to striata of saline-injected mice. In contrast, MPTP-intoxicated animals that received one injection of neutralizing antibodies against RANTES and eotaxin showed only 10–20% loss in striatal DA, DOPAC and HVA (Figure 10C–E). On the other hand, such protection was not seen in case of control IgG treatment (Figure 10C–E).

### 3.5. Functional Blocking Antibodies against RANTES and Eotaxin

**Figure 11:**
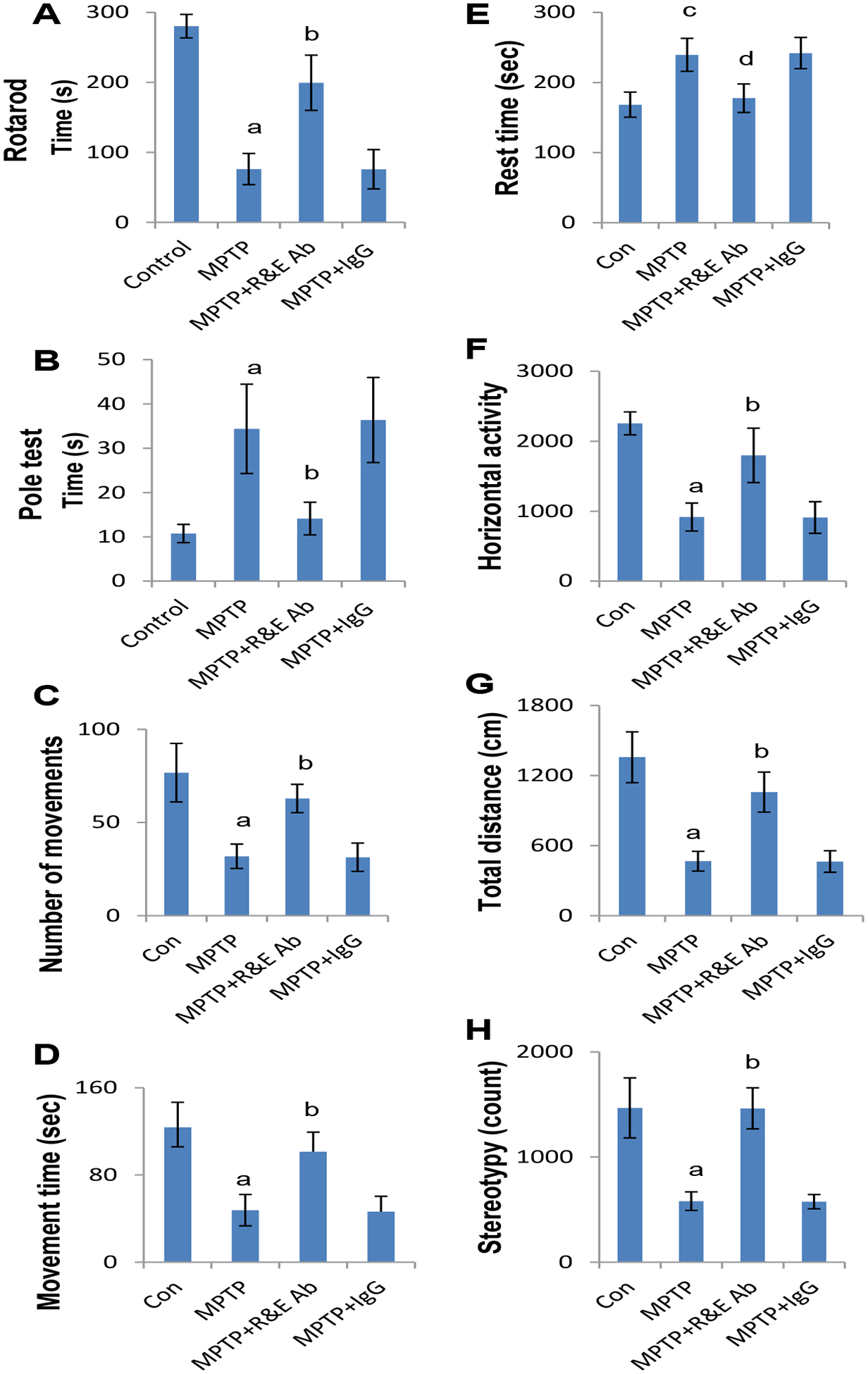
Neutralization of RANTES and eotaxin improves motor functions in MPTP-intoxicated mice. Male C57/BL6 mice (6–8-week-old) were insulted with 20 mg/kg body wt MPTP (four injections at every 2 h interval). After 2 h of the last injection of MPTP, animals were treated with the combination of 20 μg/mouse anti-RANTES Ab and 20 μg/mouse anti-eotaxin Ab via i.p. injection. After 7 d of the last injection of MPTP, mice were tested for motor functions (A, rotorod; B, pole test; C, number of movements; D, movement time; E, rest time; F, horizontal activity; G, total distance; H, stereotypy). Data are means ± SEM of nine mice per group. ap<0.001 vs control; cp<0.05 vs control; bp<0.001 vs MPTP; dp<0.05 vs MPTP.

Improve Locomotor Functions in MPTP-Intoxicated Mice The ultimate therapeutic goal of neuroprotection in PD is to decrease functional impairment. Therefore, to examine whether neutralization of RANTES and eotaxin protects not only against structural and neurotransmitter damage but also against functional impairments caused by MPTP, we monitored bradykinesia by pole test and locomotor functions by rotorod and open-field activities. MPTP insult caused a marked decrease in rotorod performance (Figure 11A), pole test (Figure 11B), number of movements (Figure 11C), movement time (Figure 11D), horizontal activity (Figure 11F), total distance (Figure 11G), and stereotypy (Figure 11H). On the other hand, MPTP insult increased the rest time (Figure 11E). However, neutralizing antibodies against RANTES and eotaxin significantly improved MPTP-induced hypolocomotion and bradykinesia (Figure 11A–H).

## 4. Discussion

PD is a progressive age-related neurodegenerative disease with unclear etiology. This disease sometimes progresses ruthlessly, leaving its victims bound to the wheelchair or dependent on caregivers. Despite intense investigations effective therapy against PD is still unavailable. Administration of a dopamine agonist or levodopa has been the standard treatment for PD. However, it is often associated with a number of side effects and unsatisfactory outcomes. Therefore, understanding the mechanism of the disease process of PD and development of effective neuroprotective therapeutic approach to halt the disease progression are of paramount importance. Here, we have seen rapid increase in RANTES and eotaxin in the nigra and serum of mice upon MPTP intoxication. Although microglia were main producers of RANTES and eotaxin in the SNpc of MPTP-intoxicated mice, we also noticed some RANTES and eotaxin in GFAP-positive astrocytes. Furthermore, increase in RANTES and eotaxin in the SNpc of postmortem PD brains as compared to age-matched controls suggest that these chemokines may play a role in the loss of nigral dopaminergic neurons in PD. Since the increase in RANTES and eotaxin was also seen in serum of MPTP-intoxicated mice, we used functional blocking antibodies to neutralize their activities in the periphery. Several lines of evidence clearly suggest that peripheral administration of functional blocking antibodies against RANTES and eotaxin reduces inflammation and protects the nigrostriatum in MPTP-intoxicated mice.

## 5. Conclusion

Our conclusion is based on the following: First, inflammation plays a role in the pathogenesis of nigrostriatal degeneration in PD patients and MPTP-intoxicated mice [18,22,23,38,39]. Accordingly, MPTP insult increased the expression of different proinflammatory cytokines (TNFα, IL-1β and IL-6) and iNOS in the SNpc. However, i.p. injection of a combination of antibodies against RANTES and eotaxin markedly decreased the expression of these proinflammatory molecules in the SNpc of MPTP-intoxicated mice. Second, as expected, MPTP intoxication led to glial activation in the nigra as evident by marked increase in expression of GFAP and CD11b, which was inhibited by treatment of antibodies against RANTES and eotaxin. Third, as observed in PD, nigral dopaminergic neurons disappeared in MPTP-intoxicated mice. But treatment with antibodies against RANTES and eotaxin protected TH-positive dopaminergic neurons from MPTP toxicity. Fourth, treatment with antibodies against RANTES and eotaxin also protected striatal TH fibers from MPTP toxicity and restored the level of neurotransmitters. Lastly, antibody treatment ameliorated functional impairment in MPTP-intoxicated mice. We did not notice any side effect (e.g. hair loss, weight loss, untoward infection etc.) in any of the mice used during the course of the study, suggesting that antibodies against RANTES and eotaxin may not exhibit any side effects.

RANTES and eotaxin are two important proinflammatory chemokines that are produced by T cells and antigen-presenting cells such as macrophages and microglia [40,41].

RANTES, a 68 amino acid-long polypeptide, is known to induce the migration and homing of classical lymphoid cells such as T cells and monocytes, and other immune cells including basophils, eosinophils, natural killer cells, dendritic cells, and mast cells [42]. Similarly, eotaxin, another small 71 amino acid-long chemokine, is capable of inducing infiltration of mononuclear cells in the site of inflammation [43]. Therefore, major function of these two chemokines is to control the homing of T cells. Earlier few studies have already reported infiltration of T cells into the nigra of MPTP mouse model and MPTP-intoxicated rhesus monkeys [8,14]. Here, we have also seen marked infiltration of CD4+ and relatively less infiltration of CD8+ T cells into the nigra upon MPTP intoxication. Interestingly, neutralization of RANTES and eotaxin strongly inhibited the infiltration of both CD4+ and CD8+ T cells into the nigra of MPTP-intoxicated mice, suggesting that MPTP insult induces infiltration of T cells into the nigra via RANTES and eotaxin.

Although whether T cell infiltration is primary or secondary to nigrostriatal degeneration is still unclear, once T cells infiltrate into the nigra, there are several direct and indirect pathways by which T cells could influence dopaminergic neurodegeneration. For example, it has been reported that the migration of antigen-specific CD4+ T cells from the periphery to the CNS generates immunocyte-microglial activities that perpetuate neuroinflammation and affect neuronal survival [22]. Earlier we have shown that effector T cells are capable of activating microglia for the production of various proinflammatory molecules via cell-to-cell contact [24,36]. This contact process involves VLA4 (α4β1) integrin of T cells and VCAM1 of microglia [24,37]. Furthermore, activated T cells may also activate microglia via CD40-CD40 ligation [7,9,11]. According to Nitsch et al, cytotoxic T cell-mediated lethal increase in neuronal calcium could be prevented by blocking both perforin and glutamate receptors [10]. In summary, we have demonstrated that MPTP intoxication leads to rapid increase in RANTES and eotaxin in the SNpc and serum of mice and that neutralization of these two chemokines protect nigral dopaminergic neurons. Although MPTP mouse model does not recapitulate all the features of PD in humans, RANTES and eotaxin are also upregulated in the nigra of postmortem PD brains as compared to control brains. Therefore, our results suggest that neutralizing antibodies against RANTES and eotaxin may have therapeutic efficacy in PD.
